# Gradient adjustment method for better discriminating correlating and non-correlating regions of physiological signals: application to the partitioning of impaired and intact zones of cerebral autoregulation

**DOI:** 10.1007/s10877-016-9913-x

**Published:** 2016-08-05

**Authors:** Paul S. Addison, André Antunes, Dean Montgomery, Ulf R. Borg

**Affiliations:** 1 Medtronic Respiratory and Monitoring Solutions, Edinburgh, Scotland UK; 2 Medtronic Respiratory and Monitoring Solutions, Boulder, CO USA

**Keywords:** Cerebral autoregulation, Regional oxygen saturation, Blood pressure

## Abstract

Cerebral blood flow (CBF) is regulated over a range of systemic blood pressures by the cerebral autoregulation (CA) control mechanism. This range lies within the lower and upper limits of autoregulation (LLA, ULA), beyond which blood pressure drives CBF, and CA function is considered impaired. A standard method to determine autoregulation limits noninvasively using NIRS technology is via the COx measure: a moving correlation index between mean arterial pressure and regional oxygen saturation. In the intact region, there should be no correlation between these variables whereas in the impaired region, the correlation index should approximate unity. In practice, however, the data may be noisy and/or the intact region may often exhibit a slightly positive relationship. This positive relationship may render traditional autoregulation limit calculations difficult to perform, resulting in the need for manual interpretation of the data using arbitrary thresholds. Further, the underlying mathematics of the technique are asymmetric in terms of the results produced for impaired and intact regions and are, in fact, not computable for the ideal case within the intact region. In this work, we propose a novel gradient adjustment method (GACOx) to enhance the differences in COx values observed in the intact and impaired regions. Results from a porcine model (N = 8) are used to demonstrate that GACOx is successful in determining LLA values where traditional methods fail. It is shown that the derived GACOx indices exhibit a mean difference between the intact/impaired regions of 1.54 ± 0.26 (mean ± SD), compared to 0.14 ± 0.10 for the traditional COx method. The GACOx effectively polarizes the COx data in order to better differentiate the intact and impaired zones and, in doing so, makes the determination of the LLA and ULA points a simpler and more consistent task. The method lends itself to the automation of the robust determination of autoregulation zone limits.

## Introduction

**Fig. 1:**
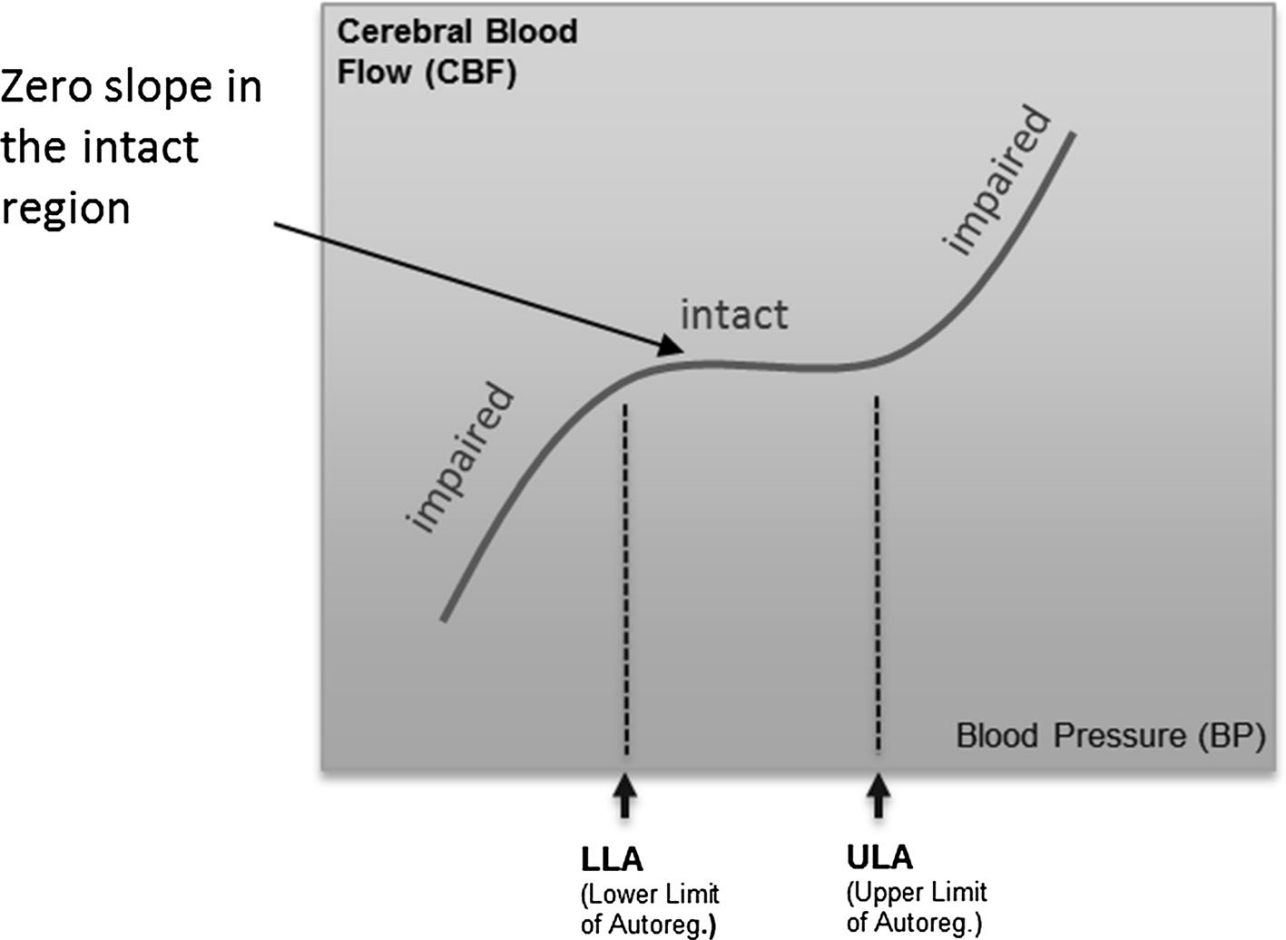
Ideal curve showing flat (zero gradient) portion in the intact region

Cerebral blood flow (CBF) is regulated over a range of systemic blood pressures (BPs) by the cerebral autoregulation (CA) control mechanism, which acts through complex myogenic, neurogenic, and metabolic mechanisms [1]. This range spans a zone of intact autoregulation from the lower limit of autoregulation (LLA) to the upper limit of autoregulation (ULA). Unregulated flow, and therefore impaired CA, occurs at the extremes of blood pressure (i.e. below the LLA and above the ULA), where cerebral vasocontrol is no longer able to adequately control vascular resistance in response to further blood pressure changes. In these impaired regions, the blood pressure drives the flow and is therefore positively correlated with it; whereas in the intact region, cerebral control of blood flow is maintained in the event of blood pressure changes and no long-term correlation between the signals exists. This behaviour is depicted in Fig. 1, which contains a schematic of the archetypal pressure-flow relationship for cerebral autoregulation. In this idealised depiction, the intact region displays a horizontal plateau indicating that no correlation between the parameters exists.

The correlation between the pressure drop across the brain-cerebral perfusion pressure (CPP)—and cerebral blood flow (CBF) may be quantified using a linear regression. The derived correlation coefficient, Mx [2], provides a measure of the coupling between pressure and cerebral flow. A common non-invasive proxy for Mx is COx: the cerebral oximetry index [3]. COx is the correlation coefficient derived from the relationship between NIRS-based regional oxygen saturation measurement (rSO_2_) and mean arterial (blood) pressure (MAP).

**Fig. 2:**
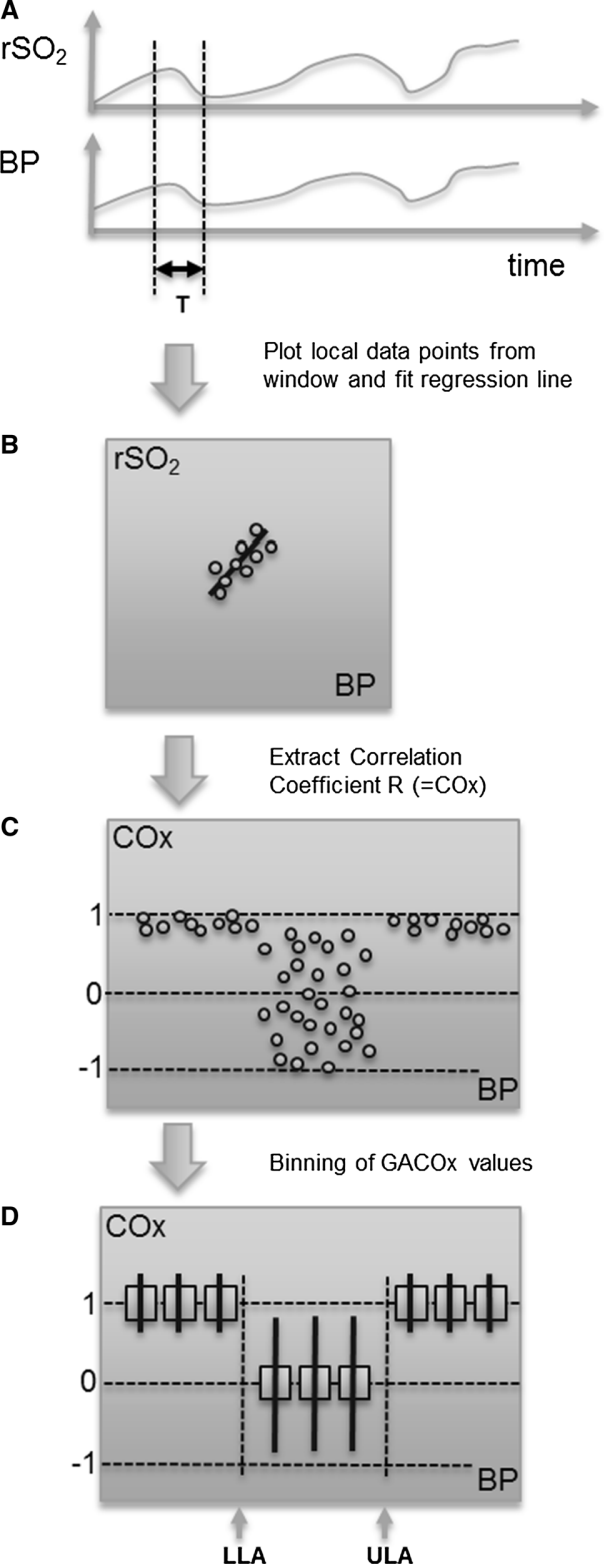
The process of generating a cerebral oximetry index (COx) plot. Note that COx values are in fact bounded by [−1 1]

The method for calculating COx is presented in Fig. 2. Plot A in the figure shows the analysis window of period T, which is run across the acquired rSO_2_ and MAP signals. The data within this window are plotted against each other (plot B). The window length is chosen to capture the characteristic periodicity of the physiological slow waves present in the blood pressure signal [4]. In practice, a time window of 300 s is usually chosen [4–6], although other window lengths have been suggested [7–10]. Linear regression of the data is then performed and the Pearson correlation coefficient calculated for each window position:

1 $$ {\text{R}} = \text{cov} \left( {{\text{X}},{\text{Y}}} \right)/\left( {\upsigma_{\text{X}}\upsigma_{\text{Y}} } \right) $$

where the vectors X and Y are the two signals under investigation, cov(X, Y) is the covariance between X and Y, and σ_X_, σ_y_ denote standard deviations: replacing X and Y by MAP and rSO_2_ defines the COx measure (similarly, using CPP and CBF results in the Mx measure. However, note that many research groups use MAP as a proxy for CPP and/or CBF *velocity* as a proxy for CBF when computing Mx). The window is then slid over the signal in incremental steps and the process repeated. In practice, this time step is around 5–10 s, which is chosen to filter out low frequency components (including cardiac and respiratory modulations). At each step, a COx value is calculated and added to an aggregated COx plot (plot C in Fig. 2), these are then used to form the COx plot often used in practice, where the values are binned in 5 mmHg increments (plot D of Fig. 2). The expectation is that a strong positive correlation exists between MAP and rSO_2_ in regions of autoregulatory impairment, hence the COx values will tend to a value of unity. Regions of intact autoregulation, however, should produce no correlation between rSO_2_ and changes in blood pressure and hence we expect the correlation coefficient (COx) to be zero. In this ideal case, we therefore expect a step change in the binned COx values when transitioning from intact to impaired regions at the LLA or ULA. In practice, however, the binned data are generally noisy and a COx threshold value somewhere between 0 and +1 is used to differentiate the correlating and non-correlating portions of the plot. Values used in studies reported elsewhere in the literature for this threshold are 0.3 [11, 12], 0.4 [13, 14] and 0.5 [3, 15].

There are, however, fundamental problems with the correlation method described above in two distinct areas: physiological and mathematical. We describe these in turn:

1. The pressure-flow curve may not contain a central ‘flat’ region of zero gradient during intact CA as depicted in the ideal case of Fig. 1. In practice, a positive gradient is often found in this region, although less steep than in the impaired region. (This case is shown schematically in Fig. 3). The idea that there is no change in the range of intact autoregulation originates from Lassen [16], who described the existence of a constant plateau for values between 60 and 150 mmHg. However, Lucas et al. [17] have reported on the pitfalls in following Lassen’s premise, and referred to a reanalysis of Lassen’s data performed by Heistad and Kontos [18] which challenges the constant plateau concept. Willie et al. [19, 20] have discussed the existence of a slope in the intact region, providing enough evidence that brain perfusion in the intact region is not constant and, more recently, Donnelly et al. [21] have also mentioned that a pressure-passive behaviour above the LLA is commonly observed. The exact mechanism that causes the functional dependence of CBF has not been determined, but there is enough evidence to challenge the assumption of zero slope in the intact region. In the case of such behaviour, the derived COx value in the intact region would also tend to unity, making it indistinguishable from regions of impaired autoregulation.

**Fig. 3:**
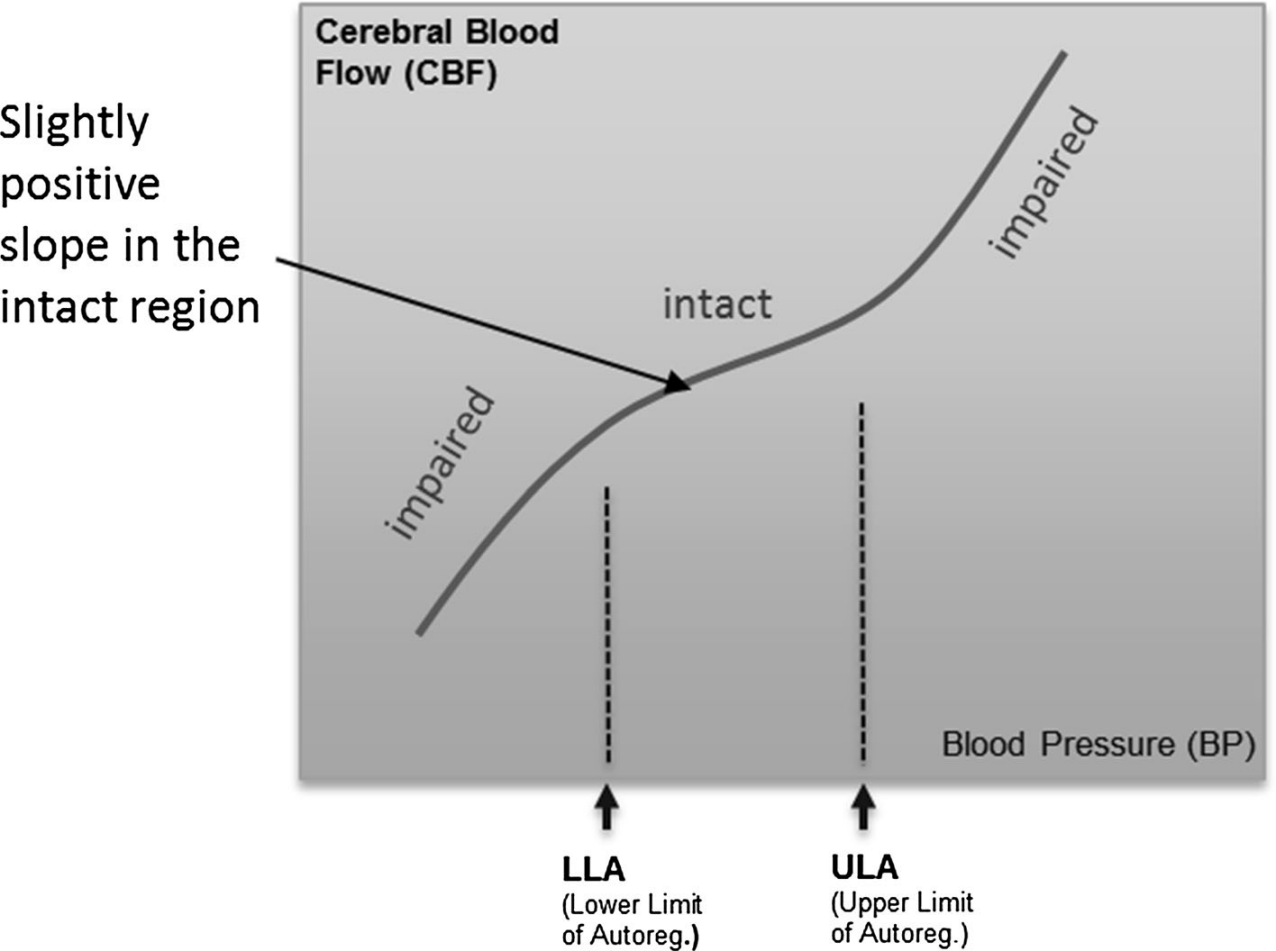
Curve showing slightly positive relationship in the intact region
2. The correlation method itself has a fundamental characteristic that makes it less suitable for clearly defining the two regions (even if an intact CA region of zero slope is present in the data). We expect a strong correlation near unity for the impaired region as MAP and rSO_2_ trend strongly with each other. However, during the intact region we expect the two signals to be decoupled. Thus, for some time periods, both signals may be trending in the same direction, showing an apparent correlation; and in other time periods the two signals may be trending in the opposite direction, showing a negative apparent correlation. Further, there may be many periods where the two have a mix of trending and anti-trending behaviour in the same time window. We thus expect the COx value during intact CA to vary from +1 to −1 randomly over time, with only the *average* value over time tending to zero (the expected distribution of the computed points prior to binning is depicted in Fig. 2, plot C). The COx method is therefore asymmetric in its behaviour according to zone. This is true for all such correlation-based methods: Mx, HVx, PRx, LDx, etc. Further, the Pearson correlation coefficient will, in theory, ‘blow up’ for the ideal horizontal relationship between rSO_2_ and BP. This can be seen from Eq. (1), where the standard deviation of the rSO_2_ signal would be zero and the index at that region become undefined (the method therefore requires some noise in the data and/or a non-horizontal flat region in the curve for this not to occur).

Taking the above into account, we propose a new method for partitioning the intact and impaired CA zones. The technique employs a gradient adjustment prior to performing the correlation analysis, which shifts the expectation from a strong value at unity and a mean value near zero for the calculated correlation coefficients (COx) to strong values at +1 and −1, corresponding to impaired and intact regions respectively. This facilitates partitioning of the data and hence aids determination of the LLA and ULA boundaries. The new method better discriminates correlating and non-correlating signal segments in data used to determine the status of cerebral autoregulation. However, the technique could find general applicability in the analysis of any other signals where regions of correlation and non-correlation require identification.

## Methods

### Gradient adjustment method

**Fig. 4:**
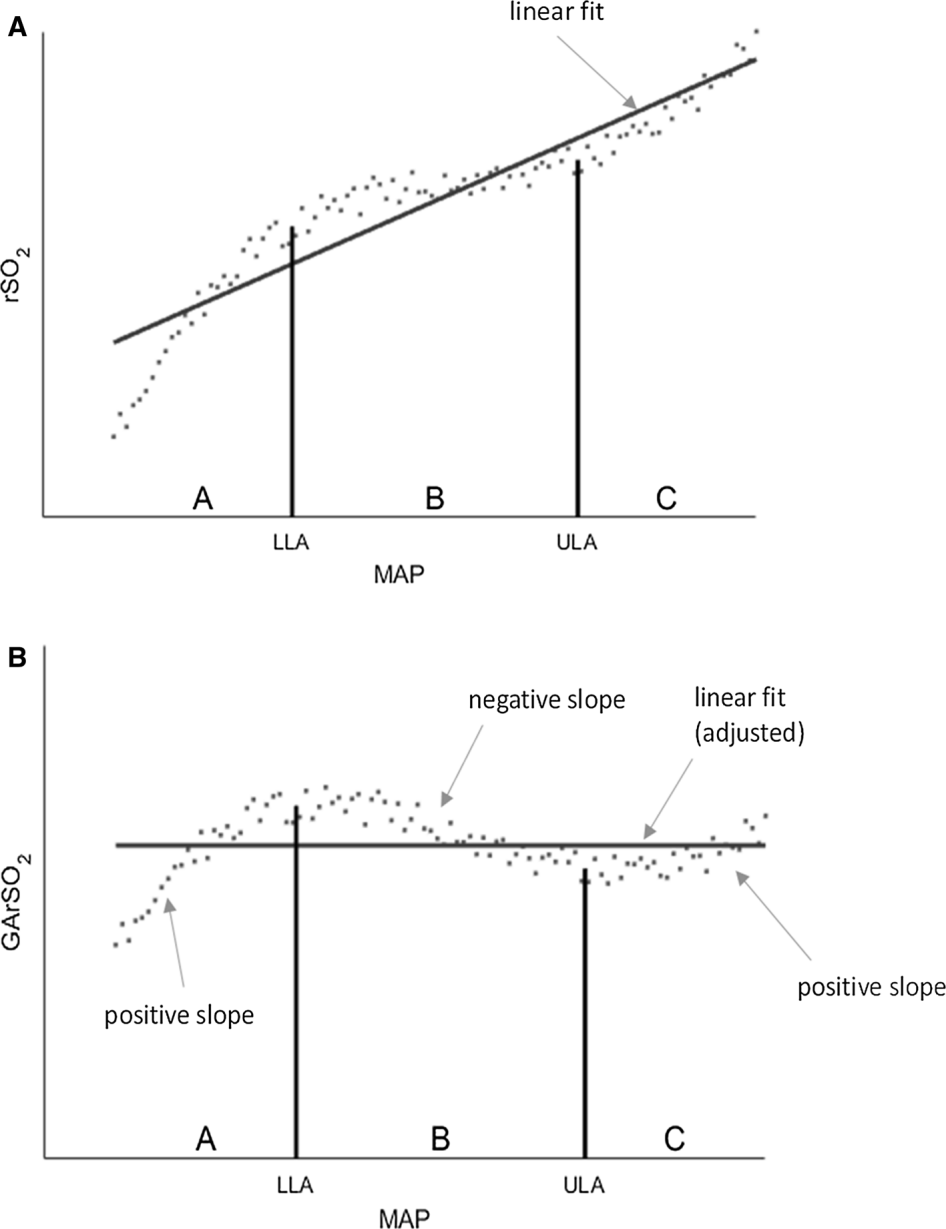
**a** Schematic rSO_2_–MAP curve shown with a flat or slightly positive slope in the intact zone (*region B*) and corresponding linear fit. **b** rSO_2_–MAP curve after applying the gradient adjustment (i.e. the GArSO_2_–MAP curve). The slope in *region B* is now negative, while in regions *A* and *C* the slope is still positive (*rSO*_*2*_ regional oxygen saturation, *MAP* mean arterial pressure)

**Fig. 5:**
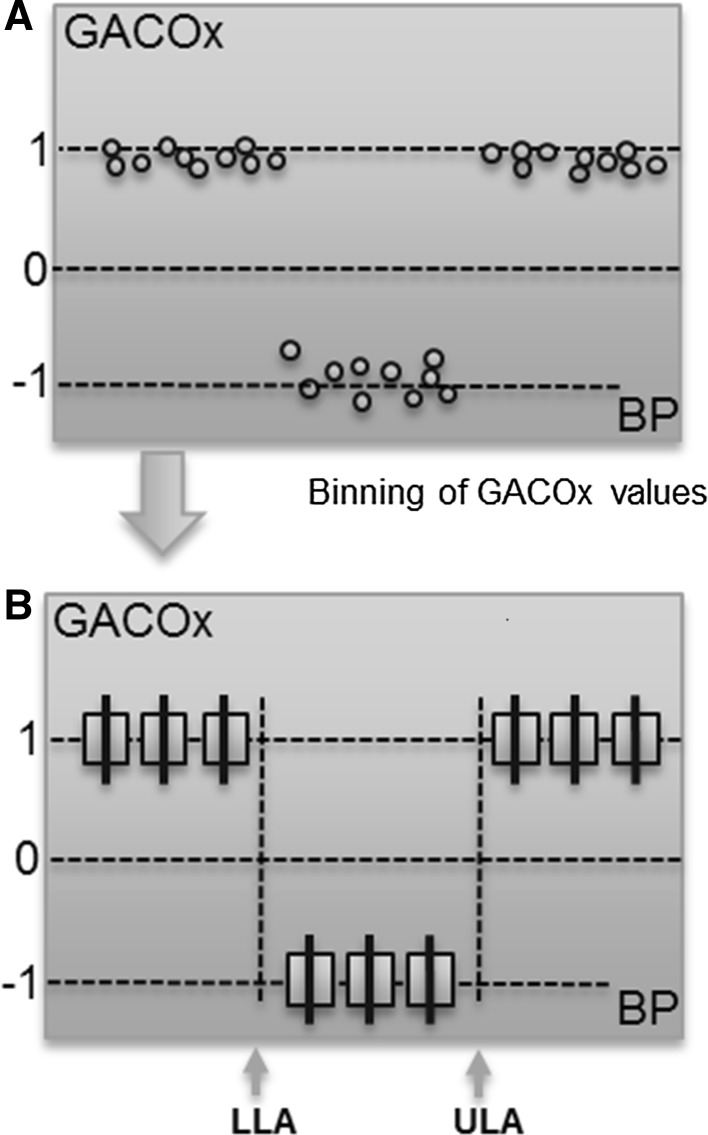
The polarization of the intact and impaired data caused by the gradient adjustment of the COx data. These plots should be compared to the traditional plots (C and D of Fig. 2) (*COx* cerebral oximetry index, *GACOx* gradient adjusted COx measure)

The gradient adjustment (GA) method significantly enhances the delineation between impaired and intact autoregulation zones in the COx plot by manipulating the underlying relationship so that the intact region is forced to have a distinct negative gradient while leaving the impaired regions with a distinct positive gradient. In the method, which is illustrated schematically in Fig. 4, we may compensate for the possibility of a zero or positive gradient in the intact region B by transforming the rSO_2_ values before calculating the correlation. This is carried out by calculating the regression line of the rSO_2_–MAP curve, shown in Fig. 4a, and subtracting the corresponding values from the rSO_2_ signal. The regression line is an equation given by

2 $$ y\left( x \right) = mx + b $$

where *y* corresponds to rSO_2_ values, *x* to MAP values, *m* is the slope of the line and *b* the intersection of the line with the y axis. The gradient-adjusted rSO_2_ is then calculated as

3 $$ GArSO_{2} \left( {x_{i} } \right) = rSO_{2} \left( {x_{i} } \right) - y\left( {x_{i} } \right) $$

where *x*_*i*_ is the MAP value for the corresponding *i*-th element in the data array and *y(x*_*i*_ *)* is the corresponding value of rSO_2_ on the regression line. As we expect the overall gradient to be greater than that of region B and less than A and C, this gradient adjustment results in a strong negative slope of the data in region B, while maintaining positive slopes in regions A and C (this is shown schematically in Fig. 4b). Thus, by using GArSO_2_ values instead of the original rSO2 values, we may calculate a gradient-adjusted COx (GACOx) plot. The GACOx method effectively polarizes the COx data in order to better differentiate the intact and impaired zones and, in doing so, makes the determination of the LLA and ULA points a simpler and more consistent task. This polarization of the data to the +1 and −1 values is shown schematically in Fig. 5.

**Fig. 6:**
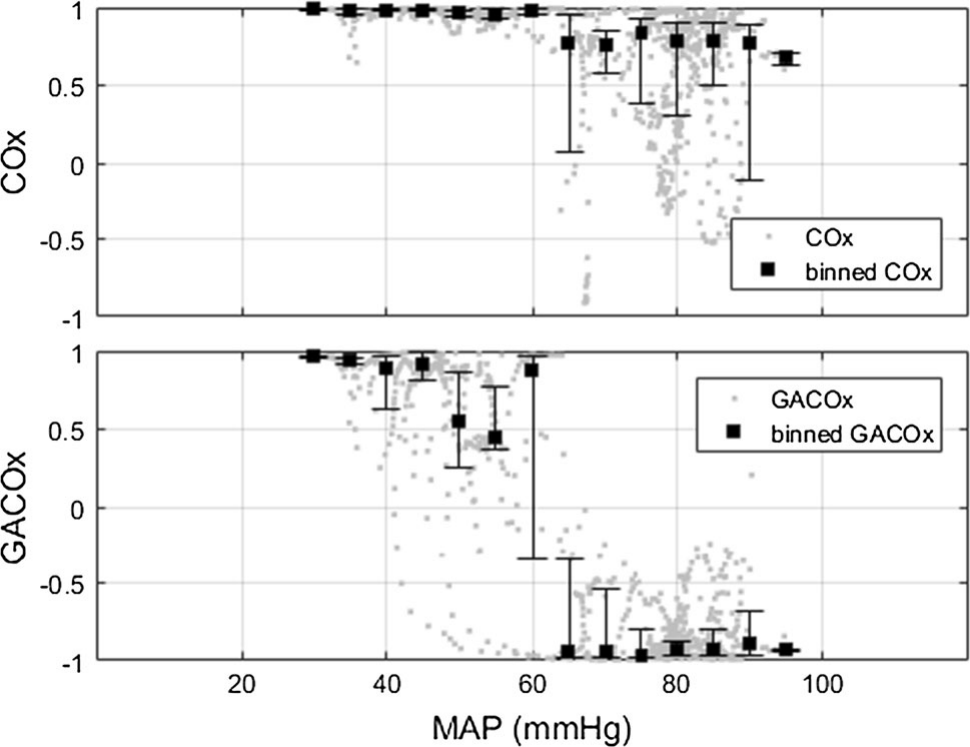
Example of a COx plot and its corresponding GACOx plot. COx and GACOx for a single trial. The *error bars* indicate the interquartile range (25–75 %) for the binned data. The *black square markers* indicate the median of the COx/GACOx values inside the bin. The bins have a width of 5 mmHg and are centred in MAP values equal to multiples of 5 (i.e. 5, 10, 15, etc.). After applying the gradient adjustment, there is a noticeable difference between the values in the intact and impaired regions (note that the original individual (unbinned) data points used to calculate the binned data are also shown in the plots) (*COx* cerebral oximetry index, *GACOx* gradient adjusted COx measure)

Figure 6 shows an example of a traditional COx plot and a GACOx plot (this comprises one of the data sets used in the study described in detail in Sect. 3 below). The traditional COx plot in the figure is typical of data with a slightly positive gradient in the intact CA region. It exhibits tight clustering towards a value of unity across the whole blood pressure range, making the LLA relatively difficult to resolve using traditional methods of analysis. However, after the application of gradient adjustment, a clearly visible separation appears between the points corresponding to the intact and impaired regions in the GACOx plot; the data tending to cluster around values of 1 and −1 respectively.

#### Comparison methods

Three reference methods were employed for the calculation of the limits of autoregulation for comparison with GACOx: manual inspection and two automated algorithms. These are described below:

1. *Manual method* The COx plots were printed out for eight data sets and three of the authors (PSA, AA, DM) were asked independently to determine the LLA from the plot. In the event of a disagreement, the manual LLA value for each animal was defined as the median of the three evaluations.
2. *Automated algorithm 1* A value of 0.5 was set as the limiting value for the transition between the low-impaired region and the intact region. The binned data was inspected, starting with the lowest blood pressure bin, moving up a 5 mmHg bin at each increment. The LLA was determined as the MAP value corresponding to the first binned COx below 0.5.
3. *Automated algorithm 2* The same procedure as Algorithm 1 but requiring at least 3 consecutive binned values below the 0.5 threshold before the LLA was determined. This was done to mitigate against false detections due to erroneous noisy data points below the threshold.

The GACOx LLAs were then determined using both a manual and automated algorithm. For the automated algorithm the threshold was simply set to zero and the first point below was determined as the LLA.

### Data and analysis

The analysis of the method presented here was performed retrospectively based on data from an animal study originally designed to investigate the characterization of cerebral autoregulation. The study consisted of a healthy porcine model (N = 8, 8 female), aged 8.4 ± 0.5 weeks (mean ± SD), with a weight of 13.3 ± 1.7 kg (mean ± SD). The protocol was reviewed and approved by the PCRS Animal Care and Use Committee. The study was conducted in GLP like fashion accordance with 21 CFR Part 58 at an Association for Assessment and Accreditation of Laboratory Animal Care (AAALAC) accredited site. The following standards in terms of appropriate use of animals for biomedical research and/or training were adhered to: The U.S. Animal Welfare Act amendment of 1976 (Title 9, Code of Federal Regulations, Chapter 1, Sub-chapter A, parts 1, 2 and 3) and the current U.S. National Institute of Health’s Guide for the Care and Use of Laboratory Animals published by the National Research Council.

Fentanyl, isoflurane, propofol and vecuronium were used as anaesthetic agents and heparin as an anticoagulant. NIRS sensors (INVOS SAFB-SM) were placed on the animals’ head between the ears. These were attached to the monitor (INVOS 5100C oximeter, 5100C-PA preamp unit (Medtronic, Boulder, CO)). NIRS cerebral signals (both raw signals and the output rSO_2_ signal) and a blood pressure signal were collected. The animal was ventilated with a tidal volume of 6–8 mL/kg, FiO_2_ was adjusted to maintain 95 % arterial saturation and PEEP was 3 cmH_2_O. Respiratory rate was adjusted to maintain end-tidal CO_2_ between 38 and 45 mmHg. Heating pads were used to maintain normal body temperature as necessary.

The signals used in the analysis were acquired during episodes of induced haemorrhagic shock and vasoconstriction using an α-agent (norepinephrine). The protocol allowed mostly for blood pressure variations which resulted in multiple crossings of the LLA. A few high pressure spikes were seen in the data but not enough to adequately define a ULA in most of the cases, hence only LLAs were considered in this study. The MAP was calculated from the raw blood pressure signal by an in-house peak detection algorithm which determines systolic and diastolic blood pressure on a beat-by-beat basis. This is averaged over 10 s and output synchronously with rSO_2_, which was acquired directly from the pre-amp signal from an INVOS 5100C regional oximeter (Medtronic, Boulder, CO).

A moving 300 s window was employed to analyse the MAP and rSO_2_ signals. This was incremented along the signals in a series of 10 s steps. The calculated COx metric values were binned in 5 mmHg blood pressure increments, and the reference LLAs calculated using the three methods described in 2.1.1. Bins with fewer than three data points were not considered as representative and were excluded from the analysis. Box plots were used to show the relative (aggregated) distributions of the COx and GACOx data either side of the determined LLAs. The difference in the median values of the COx and GACOx data was taken as a measure of the separation of the intact and impaired data.

## Results

**Fig. 7:**
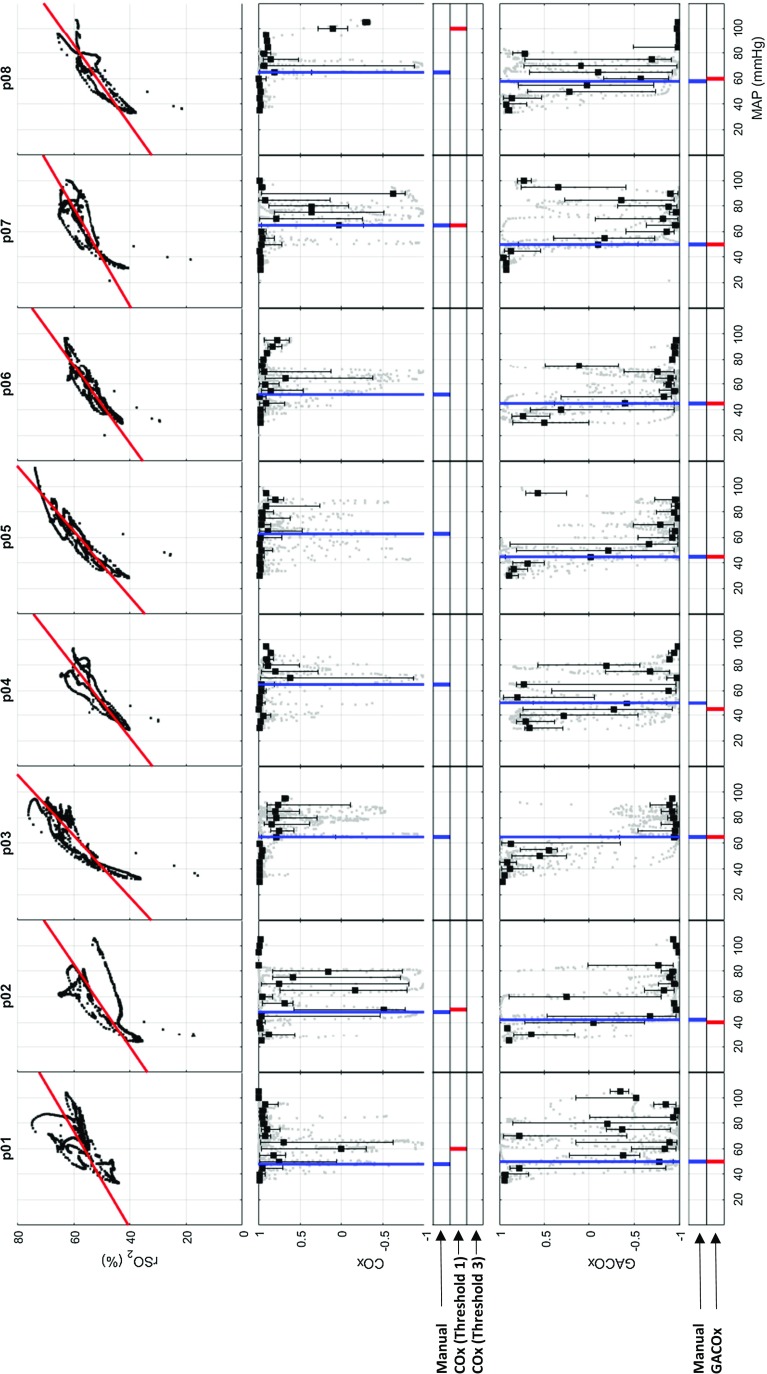
*Top row* rSO_2_ versus MAP plot with the regression line used for the gradient adjustment method. *Middle row* Traditional COx method with the manually selected LLA (drawn through the data) and an indication strip below showing the locations of the manual LLA versus the LLAs determined by the automatic COx algorithms (threshold 1 and threshold 3). *Bottom row* GACOx method with the manually selected LLA (drawn through the data) and an indication strip below showing the locations of the manual LLA versus the LLA determined by the GACOx algorithm (*COx* cerebral oximetry index, *GACOx* gradient adjusted COx measure, *LLA* lower limit of autoregulation)

Figure 7 shows COx and GACOx plots for all the animals in the study. The plots include the LLAs calculated using the manual reference method. The position of the LLAs determined from automated COx and GACOx algorithms described above are also indicated below each respective plot. The corresponding linear fits of the MAP versus rSO_2_ data, used to adjust the indices, can also be seen in the top row plots. It may be observed from the figure that all GACOx plots exhibit a clear transition from positive to negative values. This is in contrast to the traditional COx plots, where many of the transition regions are difficult to discern, hence making LLA identification problematic. A summary of the calculated LLAs using GACOx and the reference methods is provided in Table 1. It can be seen that both automated algorithmic methods, when used on the traditional COx plot, failed to identify a number of LLAs due to data not transiting below the threshold of 0.5 (in fact, the ‘Threshold 3’ algorithm failed for all cases). However, the GACOx algorithm produced LLAs for all data sets which can be seen from Fig. 7 to be very close in value to those determined manually (5 out of the 8 LLAs are in complete agreement with a mean difference of 1.25 mmHg and a maximum difference of 5 mmHg).

**Table 1:** LLAs computed using various approaches

Method	p01	p02	p03	p04	p05	p06	p07	p08
COx (manual)	48	48	65	65	63	52	65	65
COx algorithm (threshold 1)	60	50	FAIL	FAIL	FAIL	FAIL	65	100
COx algorithm (threshold 3)	FAIL	FAIL	FAIL	FAIL	FAIL	FAIL	FAIL	FAIL
GACOx (manual)	50	42	65	50	45	45	50	58
GACOx algorithm	50	40	65	45	45	45	50	60

*COx* cerebral oximetry index, *GACOx* gradient adjusted COx measure

**Fig. 8:**
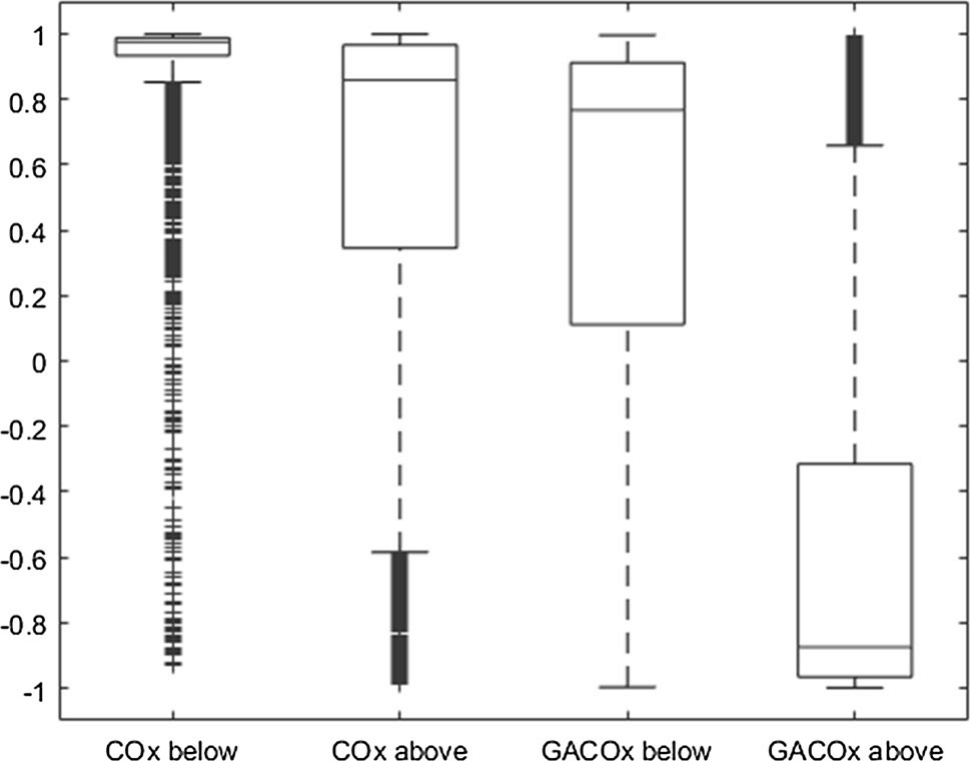
Box plot of COx/GACOx values below and above the manually derived LLAs for all data in the study. The *line* inside the *box* represent the median, while the *boxes edges* represent the 25 and 75 percentiles. The whiskers represent values 1.5 times the box length. Data points outside this limit are plotted as outliers (*COx* cerebral oximetry index, *GACOx* gradient adjusted COx measure, *LLA* lower limit of autoregulation)

Figure 8 shows the boxplots for the COx values and GACOx values split either side of their respective manually derived LLAs to highlight the differences between the data distributions in the impaired and intact regions. It may be observed that there is a significant overlap of COx data points either side of the LLA for the traditional method whereas a clear difference is apparent for the GA method. Table 2 contains the difference in the median values either side of the LLAs for the automated COx and GACOx methods for each of the eight studies. It can be seen that these range from 0.06 to 0.37 for the traditional method, whereas the GA method ranges from 1.05 to 1.80 (dimensionless units). The mean ± SD for this difference is 0.14 ± 0.10 for the traditional COx and 1.54 ± 0.26 for the GACOx.

**Table 2:** Difference of the median values above and below the manually derived LLAs for each case for the COx and GACOx data sets

Data set	COx	GACOx
p01	0.07	1.54
p02	0.16	1.76
p03	0.19	1.80
p04	0.10	1.05
p05	0.06	1.69
p06	0.09	1.33
p07	0.37	1.74
p08	0.10	1.42
Mean	0.14	1.54
SD	0.10	0.26

*COx* cerebral oximetry index, *GACOx* gradient adjusted COx measure

## Discussion

The use of traditional correlation measures for the identification of the intact and impaired regions of autoregulation has inherent physiological and mathematical limitations. Physiologically, there is an inability to differentiate between intact and impaired regions when a slightly positive slope exists in the intact region of the rSO_2_–MAP plot. Mathematically two issues exist: the asymmetric data clustering in the intact and impaired zones; and the computation of a correlation coefficient for the intact region with idealised data (with a horizontal curve) is mathematically impossible. The gradient adjusted method detailed in this paper was applied to data where the traditional COx method failed to identify an LLA in most of the data sets studied—even when a reasonably high threshold value of 0.5 was employed. A manual assessment of the data did find LLAs for all data sets, but this was critically dependent on the subjective interpretation of the reviewers and required a ‘user-defined’ threshold well above those currently used in the literature.

The gradient adjustment method proposed here mitigates against the existence of a positive slope in the intact region by altering the underlying rSO_2_–MAP relationship so that the intact region exhibits a negative slope while the impaired regions retain a positive slope. In this way, the revised GACOx plot exhibits a clear grouping of the data tending to +1 for impaired and −1 for intact autoregulation. This allows for a more robust and reliable method for detecting the decision boundary at the LLA. In fact, in many cases, the method facilitates the evaluation of otherwise undefinable limits using algorithms based on the traditional method (cf. Table 1, COx algorithms). Although thresholds were obtained manually from the traditional COx curves, these were based on the individual interpretation of the reviewer and required adjustment of perceived thresholds which were data dependent and markedly above those used in practice (0.3–0.5).

It is obvious that the GA method may be applied to other correlation-based measures of autoregulation: for example, a blood volume signal, an ICP signal or a measure of flow velocity could be used in place of rSO_2_ in order to apply this correction to HVx, PRx or Mx respectively (e.g. GAHVx, GAPRx, GAMx). The work described here is part of a wider study by the authors to investigate alternative and adjunct techniques for traditional correlation-based methods for CA. The method, for example, could prove useful for the calculation of PRx where some studies have reported difficulties in differentiating between intact and impaired regimes exhibiting very small difference (typically a PRx value as small as 0.2 is associated with dysautoregulation, compared to 0.0 for intact autoregulation) [22–24].

In previous work [25], the authors developed COx analysis methods based on data clustering. We compared the results from a Gaussian mixture model (GMM) data clustering approaches with the GA method (Table 3). The GMM method also successfully produced LLAs for all eight data sets and it can be seen that the GMM method produced results very similar to the automated GACOx method, with a RMSD (root mean square of the difference) of 3.4 mmHg between both algorithms. However, the GMM method is much more complex than the proposed gradient adjusted algorithm which, after gradient adjustment, requires only a simple threshold of zero to differentiate between intact and impaired zone data.

**Table 3:** Comparison of gradient adjustment and data clustering approaches for LLA determination

Method	p01	p02	p03	p04	p05	p06	p07	p08
GACOx algorithm	50	40	65	45	45	45	50	60
GMM algorithm	42	43	67	47	42	45	52	60

*GACOx* gradient adjusted COx measure, *GMM* Gaussian mixture model

Although we have considered the case where the intact region displays a positive slope and is therefore a confounder to the traditional correlation-based COx approach, it should be noted that distinct (paradoxical) negative slopes have also been observed in intact regions [26] for cardiac surgery patients. In this instance, the traditional COx method will tend to produce values at +1 and −1 for the impaired and intact regions respectively and hence the gradient adjustment method described here would not be necessary (although it could still prove useful in accentuating the difference in noisy data).

Note that application of the GACOx method to a real time algorithm would require a sufficient number of points to establish a linear fit with statistical meaning. It is necessary to have a minimum number of points spanning through a range of blood pressures in order for the method to be able to discern the main autoregulation function regions.

The study suffers from a number of limitations. These are described as follows:

The method requires data in both the impaired and intact regions to successfully alter the gradient so that the intact region after gradient adjustment is of a negative slope. This was not a problem for the current study, where the whole data set was analysed retrospectively. However, in practice, in order to develop a real-time implementation, this needs to be considered. As the COx curve is built up, the data may initially only be confined to one region (intact or impaired). In this case, gradient adjustment could, for example, be based on a historical knowledge of the expected gradients of impaired and intact regions.

Another disadvantage of the reported study is the lack of ULA data with which to test the method. This was due to the nature of the study. It is expected that the method will work for the identification of this upper limit given that the slope of the MAP versus rSO_2_ curve is larger in the high pressure impaired region than in the intact region. However, this will require data sets containing distinct ULAs to test the hypothesis that the method is agnostic to whether a ULA or LLA is considered.

Another disadvantage of the study was that the results could not be compared directly to an independent reference signal, as the historical data used had no useful reference signal for cerebral blood flow (as the original study was not set up to investigate autoregulation), hence no Mx could be calculated (nor the corresponding GAMx). However, the LLAs could be compared with the standard COx measure derived using various approaches, including recently developed data clustering methods. Although less than ideal, it is worth noting that many of the reference signals that are used in other studies suffer from their own issues. For example Transcranial Doppler, which is required to calculate the Mx measure, requires manual intervention, does not work for some patients and/or works intermittently, and can be very noisy [3].

## Conclusion

It is now recognised that real data behaves in a much more complex way than the traditional Lassen curve indicates, with both positive and negative correlations having been observed in practice between rSO_2_ and MAP during intact autoregulation regimes. The proposed gradient adjustment technique is simple to implement and significantly aids in the automatic extraction of the limits of autoregulation. We successfully applied the method to a pig study, with noticeable improvement in terms of successfully determining lower limit boundaries of autoregulation with values very close to those determined by manual inspection. The GACOx method appears promising as a technique for the robust automation of the identification of the limits of autoregulation.
